# A discrete choice experiment studying students’ preferences for scholarships to private medical schools in Japan

**DOI:** 10.1186/s12960-016-0102-2

**Published:** 2016-02-09

**Authors:** Rei Goto, Hiroaki Kakihara

**Affiliations:** The Hakubi Center of Advanced Research, Kyoto University, Yoshida-Honmachi, Kyoto, 606-8501 Japan; Graduate School of Economics, Kyoto University, Yoshida-Honmachi, Kyoto, 606-8501 Japan; Graduate School of Pharmaceutical Sciences, Kyoto University, 46-29 Yoshida-Shimo-Adachi-cho, Kyoto, 606-8501 Japan

## Abstract

**Background:**

The shortage of physicians in rural areas and in some specialties is a societal problem in Japan. Expensive tuition in private medical schools limits access to them particularly for students from middle- and low-income families. One way to reduce this barrier and lessen maldistribution is to offer conditional scholarships to private medical schools.

**Methods:**

A discrete choice experiment is carried out on a total of 374 students considering application to medical schools. The willingness to receive a conditional scholarship program to private medical schools is analyzed.

**Results:**

The probability of attending private medical schools significantly decreased because of high tuition, a postgraduate obligation to provide a service in specific specialty areas, and the length of time of this obligation. An obligation to provide a service in rural regions had no significant effect on this probability. To motivate non-applicants to private medical schools to enroll in such schools, a decrease in tuition to around 1.2 million yen (US$ 12 000) or less, which is twice that of public schools, was found to be necessary. Further, it was found that non-applicants to private medical schools choose to apply to such schools even with restrictions if they have tuition support at the public school level.

**Conclusions:**

Conditional scholarships for private medical schools may widen access to medical education and simultaneously provide incentives to work in insufficiently served areas.

## Background

Japan had 223 physicians per 1000 population in 2010, which was the lowest figure among the G7 countries [1]. The shortage of physicians in rural areas in particular has become a serious societal concern. Although the number of physicians increased every year from 1996 to 2006, regional imbalance worsened after 2004 [2].

A further societal problem is the shortage of physicians in some specialties. The workload for pediatricians, obstetricians, and gynecologists has increased but fewer graduates are choosing these disciplines [3,4]. This may lead to shortages of such specialists in the long run.

Few foreign physicians enter the Japanese labor market; thus, Japan’s 79 medical schools are the main source of new physicians. Among these schools, 29 (36.7%) are private. Further, in 2011, of 8923 enrollments, 3263 (36.6%) were in private schools. The Ministry of Education, Culture, Sports, Science, and Technology (MEXT) determines the maximum enrollment for each school, although the number of applicants is always more than the maximum; thus, each school has independent entrance examinations. In 2006, of 103 384 applicants, 7282 were matriculated. Indeed, medical school is one of the most popular options for high school graduates in Japan [5].

Large differences in tuition exist between public and private medical schools. In public universities, there is little difference in tuition between the medical school and other departments. In addition, the national and regional governments decide the tuition. However, the average annual tuition for 6 years’ medical education in 2011 was 6 to 13 times higher in private medical schools than public medical schools. In the former, tuition ranged from 3 730 000 yen (US$ 37 300, given US$ 1 = 100 Japanese yen) to 8 200 000 yen (US$ 82 000), which is 5 590 000 yen (US$ 55 900) on average. In the latter, tuition ranged from 583 000 yen (US$ 5830) to 633 000 yen (US$ 6330).

Two of the 29 private medical schools were founded for specific reasons. Jichi Medical School was founded in 1972 to produce rural physicians. Students’ tuition and living expenses are paid by local government; in return, students are required to work for public medical institutions in their home prefectures for 9 years after graduation [6]. The University of Occupational and Environmental Health educates industrial physicians [5]. Here, students receive a scholarship covering two thirds of the total tuition in exchange for 9 years’ service as an industrial physician. Recently, the Japanese government created a budget with quotas to train new physicians at private medical schools. Such physicians must then work in rural settings for an obligatory period [7,8]. Only 3 of the 29 private medical schools provide subsidies for students [9]. These subsidies are paid by the government. Further, the quotas are modest compared with those for public medical schools. Subsidy amounts and obligations vary widely across the schools. Conditional scholarships for specific specialties are uncommon. To our knowledge, there is no scientific research that studies the impacts of these subsidies on students’ career choices even in the short term.

The role of public medical schools differs to that of private schools. In Japan, the system of higher education is hierarchical, with higher academic status usually accorded to academically elite universities that were founded by the government [10]. Twelve universities with medical schools appear in the Times Higher Education’s 2011–2012 World University Rankings, and 11 of these are public universities [11]. Thus, public universities tend to put more stress on research than on education and are limited in their capacity to train rural physicians and physicians with specialties that are in insufficient supply. In contrast, private medical schools put more resources into education in general. They are important potential resources of medical education for deficient fields.

Medical education in private medical schools is unaffordable to most students and their families in Japan. Medical school begins immediately after graduation from high school. Student loans are not popular. The average annual income of households with university students is around 8 000 000 yen (US$ 80 000) [12]. The financial barrier to private medical school education restricts human resource development. Also, private medical school graduates want a high income to recoup the high cost of their education and will therefore avoid jobs with adverse conditions.

One option for solving these problems is to provide scholarships to private medical schools in exchange for service as medical practitioners in insufficiently served regions and specialties for certain periods of time. These scholarships may widen access to medical education and simultaneously provide incentives to work in insufficiently served areas.

In this paper, students’ preferences for conditional scholarships to private medical schools are analyzed. We consider two obligations: services in rural locations and in shortened periods of specialty work. Our purpose is to estimate the demand for this type of scholarship and examine its potential effect on the unbalanced distribution of physicians in Japan.

## Methods

We conducted a questionnaire survey of students preparing to take the entrance examination for medical school. Such students participate in out-of-school preparatory activities such as private cram school sessions. If high school graduates are not admitted to the university of their choice, they may spend one or more additional years preparing. Such students are known as *ronin*, a word that was once used to describe lordless samurai [13]. Graduates of non-medical university departments (*sai-juken* students) are another source of medical school applicants. This survey was conducted in a private cram school chain where students were requested to fill out the questionnaire. Information such as age, gender, the medical schools of his/her choice, number of siblings, and occupation of parents, together with information related to a discrete choice experiment (DCE) described below, was gathered. This research is exempted from the requirement of ethics approval because this research is neither concerned with pathology, etiology, prevention, diagnosis nor treatment of particular diseases [14].

### The DCE on willingness to enter private medical schools

With a DCE, the extent to which an individual values a good or a service can be evaluated by the choice of hypothetical scenarios. This technique has been applied in health care settings, and the outcomes have revealed that DCE results have internal validity and consistency [15].

A number of DCE studies have investigated the job preferences of health care providers [16,17]. These studies found that non-pecuniary incentives are important determinants, such as out-of-hours work [18] and sufficient opportunities for professional development [17,19,20], as well as financial incentives. However, studies about medical students’ preferences are limited although some researchers have found that better working and living conditions are important factors in persuading medical students to work in the rural areas of developing countries [21,22].

To choose proper attributes, group discussions with four medical students and two physicians were held. All participants thought that school location was important. Participants from public medical schools stated that higher tuition is a significant barrier to private medical schools. Moreover, several of their high school classmates relinquished the idea of going to medical school once they failed exams to a public medical school. Participants also indicated that scholarships to private medical schools were an effective way to increase entrants despite career choice restrictions. It was interesting that those from private medical schools had different opinions. Some of their classmates wanted to enter family-owned clinics. Thus, restrictions about their career choice were inappropriate.

From the group discussions, it was evident that there may be variations in preference between public medical school students/applicants and private ones. To establish the differences in characteristics between respondents willing to apply only to public medical schools and those willing to apply also to private medical schools, probit estimation concerning the choice of schools was conducted. The dependent variable was dichotomous (students applying only to public medical school equal to 1, otherwise equal to 0).

Finally, five attributes were selected concerning tuition, location, commutability, obligations after graduation, and the length of such obligations.

### Annual tuition

The average annual tuition after receiving a scholarship ranges from 0 yen (no out-of-pocket payment) to 1 800 000 yen (US$ 18 000), which is half of the tuition in the lowest present case. The other tuition levels are 600 000 yen (US$ 6000) at national universities and 1 200 000 yen (US$ 12 000) at science and technology schools in private universities.

### School location

The average Japanese student prefers urban universities. When the school is far from home, students must live alone and their living costs increase accordingly. Sharing a flat and living in a dormitory is uncommon in Japan. Preferences for independence and daily living activities influence whether students choose schools to which they can commute from their homes.

### Conditional scholarships with obligations: rural services and/or predetermined specialties

Some medical schools offer scholarships in exchange for obligations. We considered two obligations: services in rural locations and services in predetermined specialties. The length of the obligation may discourage students from choosing a school because they have no choice about their careers. However, the impact of the obligation’s form may vary. Four service obligation options may be available: no obligation, a service provided to a specific rural region, a service limited to a specific specialty, and a service that is provided to a specific rural region and limited to a specific specialty. In Japan, compulsory initial postgraduate training lasts for 2 years. The length of advanced training for specialties is at least 3 years. Service obligations may be for 5, 7, or 9 years. Obstetrics, gynecology, and pediatrics are named as examples of specialties to which obligations may apply. Table 1 summarizes the attributes and levels included in the DCE.

**Table 1:** **Attributes and levels used in DCE**

**Attributes**	**Levels**	**Level coding**
Average annual tuition after receiving scholarship	0	0
	600 000 yen ($6 000)	60
	1 200 000 yen ($12 000)	120
	1 800 000 yen ($18 000)	180
Location of medical school	Suburban or rural area	0
	Urban area	1
Possibility to commute from his/her home	Possible	0
	Impossible	1
Postgraduate obligation for local regions	Not present	0
	Present	1
Postgraduate obligation for specific specialties	Not present	0
	Present	1
The length of postgraduate obligation	0 (no obligation)	0
	5 years	5
	7 years	7
	9 years	9

### Design of the DCE

Since the number of profiles becomes unwieldy if all possible combinations are considered, we adopted an orthogonal planning method [23] and excluded inconsistent combinations of levels, such as “no obligation” and “7 years’ obligation.” Using SPSS software, an orthogonal main effects design was sampled from the full factorial to produce 16 scenarios. These were split into two versions of eight scenarios such that all attribute levels appeared equally in each version. Respondents were asked to indicate their willingness to enter private medical school under each of eight different conditions for receiving a scholarship.

**Figure 1:**
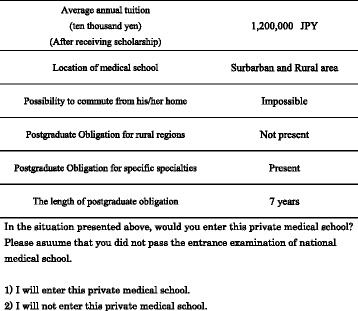
Sample question.

Respondents were asked to assume a situation in which they are accepted by just one private medical school. Most scholarships in such schools do not allow students to apply to more than one school. Thus, a binary choice framework was adopted. Figure 1 depicts the scope of the representative question that covered profiles and attributes.

### Other variables

In addition, we used age, gender, and a dummy variable (which is coded 1 if the student’s parent is a physician) as independent variables.

### Estimation

The multilevel independent variables shown in Table 2 are age, gender, occupation of parents, and status of the student. To analyze data of repeated binary-dependent variables, we used a random logit model. This DCE originally assumed that all samples have the same utility function. However, those who consider applying to private medical schools may have different preferences from those who do not. Thus, we used the log likelihood ratio (LR) test to check whether there was any overall difference in preference for medical school between the two groups [24].

**Table 2:** **Descriptive statistics of the samples**

	**Whole sample** **(** ***n*** **= 347** **)**
Age	19.3 ± 2.4
Female	115 (33.1%)
Status of students	
High school students	28 (8.1%)
Ronin	278 (80.1%)
Sai-juken	41 (11.8%)
Location of preparatory school	
Hokkaido	25 (7.2%)
Tohoku	20 (5.8%)
Kanto	155 (44.7%)
Hokuriku	27 (7.8%)
Chubu	45 (13.0%)
Kinki	9 (2.6%)
Chugoku	39 (11.2%)
Shikoku	0 (0%)
Kyusyu	27 (7.8%)
Founder of high school	
National	20 (5.8%)
Local government	166 (47.8%)
Private	161 (46.4%)
Occupation of parent	
Public servant	50 (14.4%)
Company executive	26 (7.5%)
Employed	96 (27.7%)
Teacher	15 (4.3%)
Medical practitioner	47 (13.5%)
Hospital physician	32 (9.2%)
Health professional other than physician	22 (6.3%)
Self-employed	46 (13.3%)
Farmer and fishery	0 (0%)
Legal professional	1 (0.3%)
Freelance	6 (1.7%)
Part-time worker	4 (1.2%)
Unemployed	2 (0.6%)
First-choice medical school	
National	242 (69.7%)
Local government	39 (11.2%)
Private	38 (11.0%)
Special medical school (Jichi and Sangyo Medical School)	12 (3.5%)

The percentage is shown within each category.

Choice probabilities for entering private medical school can be simulated as a function of the amount of scholarships. We conducted the simulation under two extreme situations: no postgraduate obligation (case 1) and 9 years’ obligation for service in specified specialties in certain rural regions (case 2).

Then, we briefly computed how much the total amount of scholarships and the number of physicians reserved for the underserved area will change according to the obligations. We assumed that a conditional scholarship is provided for 326 students (approximately 10%) from a total 3263 enrollments and that the proportion of non-applicants to private schools is the same with this sample.

We used Stata 13 (Stata Corp. College Station, Texas, USA) for the estimation and adopted 0.05 as the significance level for each estimate.

## Results

The questionnaire respondents were 374 students who participated in a preparatory school’s spring lectures and considered medical school as their first choice. Of these 374 students, 347 (92.8%) answered all the questions. Table 2 lists the descriptive statistics for the variables in our analysis.

This survey was conducted from April to May, 2009, a few months after entrance examinations. Among students preparing for examinations in 2010, the third-grade high school students tended to avoid the most difficult part of the preparation. *Ronin* and *sai-juken* students gave assurances that they would enter medical school if they passed the examination. The sample included 28 (8.1%), 278 (80.1%), and 41 (11.8%) high school, *ronin*, and *sai-juken* students, respectively. Although this sample may have contained fewer high school students than the group that actually entered medical school, the students in the sample did confirm their willingness to enter medical school and to honestly and correctly answer the questionnaire.

The students were from all over Japan except Shikoku, the smallest of the four main islands. The most common occupations of the students’ parents were employed workers (27.7%), followed by physicians (22.7%), and public servants (14.4%). The first choice of 80.9% of respondents was public medical schools, and only 11.0% chose private medical schools. The intention of 65.4% of all respondents was to apply only to public medical schools. Around two thirds of students were under consideration as candidates by public medical schools.

Table 3 shows the results of a probit estimation concerning the choice of schools to which the students applied. Age, gender, and status of students are not associated with medical school choice. However, the occupations of parents have significant effects on their choice. Students whose parents are public servants are significantly more likely to apply only to public medical schools, while students whose parents are medical practitioners or health professionals other than physicians are significantly more likely to apply to private medical schools.

**Table 3:** **Probit estimation results concerning the choice to apply only to public medical schools**

	**Estimates**	***p*** **value**
Age	0.041 6	0.406
Female dummy	0.062 4	0.709
Status of students		
Ronin (*n* = 278)	0.254 2	0.394
Sai-juken (*n* = 41)	0.178 7	0.714
Occupation of parent		
Public servant (*n* = 50)	0.675	0.016
Company executive (*n* = 26)	−0.126 6	0.668
Teacher (*n* = 15)	−0.362 5	0.312
Medical practitioner (*n* = 47)	−1.125 5	0.000
Hospital physician (*n* = 32)	−0.467 4	0.076
Health professional other than physician (*n* = 22)	−0.739 5	0.015
Self-employed and others (*n* = 53)	−0.138 3	0.567
Part-time worker (*n* = 4)	−0.153 1	0.826
Unemployed (*n* = 2)	−1.096 6	0.287
Constant	−0.423 5	0.641
McFadden’s *R*-squared	0.117 2	
*n*	347	

The dependent variable is dichotomous (students applying only to public medical school equal to 1, otherwise equal to 0). “High school student” is the reference variable in “Status of students.” “Employed” is the reference variable in “Occupation of parent.” “Self-employed and others” includes “Self-employed,” “Legal professional,” and “Freelance”.

Table 4 shows the estimation results from the DCE. Positive estimates denote that these attributes elevate the probability of entering private medical school.

**Table 4:** **Estimation results of the DCE**

	**Whole**		**Non-applicants to private school**		**Applicants to private school**	
	**(** ***n*** = 347**)**		**(** ***n*** **= 227** **)**		**(** ***n*** **= 120** **)**	
	**Coefficient**	***p*** **value**	**Coefficient**	***p*** **value**	**Coefficient**	***p*** **value**
Average annual tuition	−0.011	0.000	−0.014	0.000	−0.006	0.000
Urban location	0.206	0.154	0.086	0.658	0.428	0.005
Impossibility of commuting from home	−0.243	0.099	−0.304	0.122	−0.185	0.230
Obligation for specific specialties	−0.489	0.000	−0.4	0.026	−0.724	0.000
Obligation for rural regions	0.212	0.192	0.322	0.131	0.01	0.958
Length of obligation	−0.215	0.000	−0.203	0.000	−0.276	0.000
Age	0.054	0.004	0.072	0.003	0.039	0.223
Female dummy	0.101	0.297	0.042	0.726	0.238	0.180
Physician’s child	−0.17	0.109	−0.381	0.018	0.043	0.784
Constant	1.59	0.000	1.538	0.004	1.667	0.013
Log likelihood	−1 535.99		−962.84		−531.06	
McFadden’s *R*-squared	0.143		0.167		0.158	
Number of observations	2 776		1 816		960	

The likelihood of entering private medical school is significantly decreased by high tuition (*p* < 0.001). With regard to variables concerning obligations, longer obligations (*p* < 0.001) and obligations to serve in specific specialty areas (*p* < 0.001) significantly decrease the likelihood of entering medical schools, while obligations to serve in rural regions have no significant effect (*p* = 0.192). Older students are significantly willing to enter private medical schools. The constant term is significant and positive.

Preference differences are significant between the two subsamples (chi-squared: 84.2, *p* < 0.001). Overall, there is a difference in preferences between those who consider applying to private medical schools and those who do not.

The DCE result for each subsample shows that urban location is positively significant for those thinking of applying to private medical school and not significant for those who are not. The constant term is significant and positive for both subsamples.

**Figure 2:**
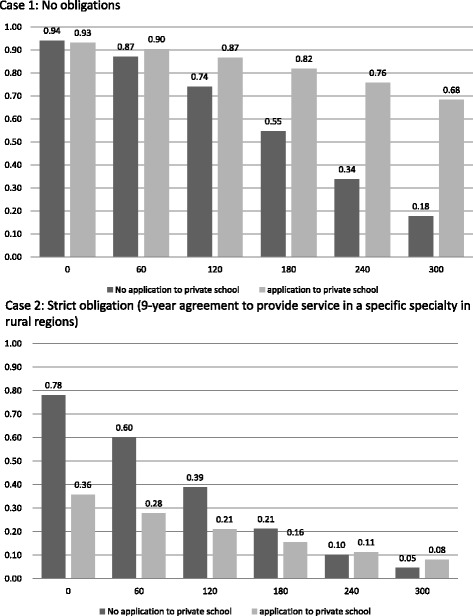
Tuition and the probability of entering private medical school. Case 1: no obligations. Case 2: strict obligation (9-year agreement to provide service in a specific specialty in a rural region). Note: The change in the probability of entering private medical schools with regard to tuition is estimated. In both cases, DCE variables, except for tuitions and obligations, are fixed at the sample mean.

Using these estimates, we simulated the change in the probability of entering private medical schools with regard to tuition by changing the obligation assumptions. Figure 2 shows the relationships between tuition and the probability of going to private medical schools for two subsamples. All independent variables, except for tuitions and obligations, are fixed at the sample mean.

In case 1, without any restrictions to their postgraduate careers, almost all students enter private medical schools when tuition is 600 000 yen (US$ 6000), which is the public medical school tuition level. For non-applicants to private medical schools, the probability drops sharply to less than 40% as tuition increases to 2.4 million yen (US$ 24 000), the lower limit of private school tuition, indicating that tuitions are indeed a barrier to the choice of private medical schools as an alternative. However, the probability remains around 60% for students who consider applying to private medical schools even when tuition is 3 million yen (US$ 30 000). In case 2, compared with case 1, the enrollment probability decreases in both samples. If all tuition is waived, 78% of non-applicants to private medical schools are willing to enroll even under case 2 service obligation conditions, although only 36% of those who considered applying to private medical schools are now willing to enter.

We briefly computed the total amount of scholarships and the number of physicians reserved for the underserved areas according to the obligations. The assumption here is that a conditional scholarship is provided for 326 students (approximately 10%) from a total of 3263 enrollments to private medical schools in 2011. In addition, the proportion of non-applicants to private medical schools is assumed to be the same with this sample. Thus, 212 students (65%) are non-applicants to private medical schools and 114 (35%) are applicants from among all scholarship candidates. When we use the results of Fig. 2, 88 (41.5%) non-applicants and 9 (7.8%) applicants accept this condition if the post-scholarship annual tuition is set at US$ 6000 in exchange for 9 years of service in specified specialties in certain rural regions (case 2). In this case, total scholarship costs are US$ 6 936 100, which is calculated from the number of those willing to accept the conditional scholarship times (average annual tuition of private medical school minus post-scholarship annual tuition). Table 5 shows the number of those who are willing to accept conditional scholarships, the total annual scholarships, and the amount of scholarships needed to secure 1 year’s physician labor power when we change the obligations. As the duration of an obligation becomes longer, so the number of those willing to accept conditional scholarships decreases. The figure for total annual scholarships is at its lowest for a 9-year obligation. However, 5 years increases physician labor power with a higher total amount of scholarships.

**Table 5:** The number of physicians secured and total amount of scholarship in exchange for predetermined specialties in rural areas

Duration of obligation	After-scholarship annual tuition ($)	The number of those willing to accept the conditional scholarship			Total annual scholarship ($)
		Non-applicants	Applicants	Total	
9 years	6 000	107	32	139	6 936 100
	12 000	63	24	87	3 819 300
	18 000	32	18	50	1 895 000
7 years	6 000	128	31	159	7 934 100
	12 000	83	24	107	4 697 300
	18 000	45	18	63	2 387 700
5 years	6 000	147	61	208	10 379 200
	12 000	104	51	155	6 804 500
	18 000	61	40	101	3 827 900

Conditional scholarships are assumed to be provided for 326 students (approx. 10%) from a total of 3263 private medical school enrollments in 2011. Also, the proportion of non-applicants to private medical schools is assumed to be the same with this sample. Thus, 212 students (65%) are non-applicants to private medical schools and 114 (35%) are applicants among all scholarship candidates. The number of those willing to accept scholarships is calculated from the probability of entering private medical schools in the event of scholarships in exchange for 9 years of service in specified specialties in certain rural regions. Each probability is estimated when we change the duration of the obligation and tuition using estimates from the DCE. The probability in specific cases is shown in Figure 2. Total annual scholarships are calculated as the number of those willing to accept them (average annual tuition of private medical schools minus post-scholarship annual tuition).

## Discussion

Conditional scholarships or scholarships with a service requirement have been implemented to increase the number of physicians in underserved areas. Existing studies show that these measures have placed substantial numbers of physicians in such areas [25]; however, the factors that drive students to participate in the scholarship program are yet to be established. In particular, participation in the program may depend on the amount of the scholarship and each student’s financial constraints. Information about the responsiveness to scholarships of varying amounts is important in countries where students must pay high tuition for their education.

In Japan, the government is considering measures to increase the total number of physicians [26]. However, an unbalanced distribution of physicians is still a societal problem. Although several scholarships are available for students who wish to work in rural regions, most of these are limited to public medical schools. Tuition at private medical schools, which represent around 40% of medical schools in Japan, are at least five times higher than those at public medical schools. This financial barrier limits access to private medical schools, particularly for students from middle- and low-income families. One way to reduce this barrier and improve the balance of distribution of physicians is to offer conditional scholarships to private medical schools. This paper investigates factors associated with the willingness of students to enroll in private medical schools when some postgraduate obligations are imposed in exchange for a decrease in financial burden. The major findings are as follows.

First, the probability of attending private medical school is significantly decreased by high tuition, postgraduate obligation to provide a service in specific specialty areas, and a long obligation period. Second, an obligation to provide a service in rural regions has no significant effect on this probability. Third, to motivate non-applicants to private medical schools to enroll in such schools, a decrease in tuition to around 1.2 million yen (US$ 12 000) or less, which is twice that of public schools, is found to be necessary. Fourth, non-applicants to private medical schools will choose to apply to private medical schools even with restrictions if given tuition support at the public school level.

High tuition deters students from applying to private medical schools. A difference in willingness to pay and ability to pay for medical education in private medical schools exists between potential applicants and non-applicants. Thus, there is a financial barrier to medical education at private schools. This barrier may increase the difficulty in recruiting qualified students to private medical institutions [27].

An obligation to provide a service to rural regions has no significant effect on the decision to enter private schools *ceteris paribus*. Before entering medical school, students may not care about where they live during the beginning of their careers as physicians. The lack of information is a possible explanation for this view. For example, students may not know about the obstacles they may face with rural services. With conditional scholarships, students commit to participation in a program before their medical education begins. If their preferences change during their education, some may leave before the start of the service obligation. In this regard, the contract compliance rate for graduates of Jichi Medical University is reported to be 97% [7]. The settlement rate after satisfaction of the obligation is also high (69.8%) [28]. Moreover, it is worth noting that the curriculum of this medical school tries to encourage students to engage in rural services and cooperate proactively with host prefectures. To achieve a high settlement rate in the long run, medical schools will have to pay more attention not only to offering scholarships but also to investment in the educational system.

Respondents are not reluctant to accept work in rural areas because they may know about public support for physicians in such locations. For example, many prefectures have procedures to dispatch substitute doctors. In addition, during the beginning of their careers, specialist education is secured for physicians. Indeed, graduates from the Jichi Medical School can have clinical training in a general hospital in a prefectural capital during service obligations.

An obligation to provide a service in specialty areas is a strong disincentive to apply for medical school. Under the current medical education system, physicians choose a specialty after 2 years of postgraduate training. Thus, students may be confused by limitations placed on their freedom of specialty choice. Figure 2 shows that non-applicants to private medical school accept this limitation when tuition is low enough. However, during preclinical and clinical training, medical students construct their professional identity through a process of medical socialization [29]. Thus, medical students receiving this kind of scholarship will need to cultivate motivation for the specialty.

The positive constant term of the DCE may show an overall willingness to enter medical schools. If respondents reject a scholarship, they must choose between spending one more year as *ronins* or giving up the idea of going to medical school. Thus, the situation whereby they are accepted by just one private medical school may encourage them to accept entry to a private school. In this DCE, respondents are encouraged to assume that they are only accepted by one private medical school. This assumption may force them to choose to go to private medical school. The inclusion of an option such as “choose none” could avoid a forced choice in which the strength of preference for entrance is falsely increased [30].

The government can gather a physician labor force for underserved areas by using such a scholarship program. It is interesting that non-applicants are more willing to do obligatory service in an underserved area and/or specialty than applicants. Indeed, a conditional scholarship may have an impact on the distribution of physicians from different financial backgrounds. Students from poorer families could go to rural locations and choose unpopular specialties, while those from richer families could enter medical practice in more popular areas and specialties. However, conditional scholarships provide access to medical education for students who would not have the means to fund an education without a financial incentive [25]. This effect by which a country’s educational system could absorb additional students may be balanced against the distributional issue.

This research has several limitations. First, the sample has a generalizability issue. The data were taken from class attendances of a compulsory subject for medical school applicants. However, the sample included fewer high school students because the survey was conducted almost 1 year before the entrance examination. Thus, there were too many *ronin* and *sai-juken* students. It is difficult to conduct a survey just before examinations in order to correct this problem.

Second, attributes and their levels have been set after group discussions with medical students and physicians. Baker et al. used Q methodology to arrange potential attributes proposed by group discussions [31]. Recently, the importance of qualitative work for attribute development has been revisited [32–34]. For example, group discussion is inappropriate if the subjects are too sensitive to discuss. More careful attribute development through collaboration with researchers with high qualitative research skills may reveal different factors from those analyzed in this research.

Third, we only investigated the main effects of attributes in the DCE. We do not know whether the preference for one attribute’s level is dependent on that of a second. For example, preferences for an obligation may depend on how long the obligation lasts. Using an interaction effects model is therefore important in order to know how a decision is related to a complex interaction of factors that either reinforce or contradict each other.

Fourth, we asked respondents to assume that they have been accepted by just one private medical school. This assumption implicitly removes some students from being candidates for scholarships. For example, those who have been accepted by another private medical school may change school and choose a conditional scholarship if tuition is sufficiently low. It would be possible to gather more information if we consider more flexible substitution patterns.

Fifth, this survey analyzes only students’ preferences and not those of their parents. Discussions with parents may determine whether a student preference actually reflects the true choice of medical school. Finally, only demand-side preference was analyzed here. To endow a scholarship program, a long-term commitment to a budget and measures to maintain compliance with obligation requirements will be needed.

## Conclusions

Financial barriers limit access to medical education in private medical schools, particularly for students from middle- and low-income families. Also, a shortage of physicians in rural areas and in some specialties is a societal problem in Japan. Conditional scholarships for private medical schools organized and funded by the government can reduce this barrier and improve the balance of distribution of physicians. This measure will therefore expand opportunities for medical education and simultaneously reduce the distribution problem of physicians.
